# Real-world effectiveness of anti-interleukin-23 antibodies in chronic plaque-type psoriasis of patients from the Austrian Psoriasis Registry (PsoRA)

**DOI:** 10.1038/s41598-022-18790-9

**Published:** 2022-09-05

**Authors:** T. Graier, W. Weger, C. Jonak, P. Sator, C. Zikeli, K. Prillinger, C. Sassmann, B. Gruber, W. Saxinger, G. Ratzinger, C. Painsi, A. Mlynek, N. Häring, B. Sadoghi, H. Trattner, R. Müllegger, F. Quehenberger, W. Salmhofer, Peter Wolf

**Affiliations:** 1 Department of Dermatology and Venereology, Medical University of Graz, Auenbruggerplatz 8, 8036 Graz, Austria; 2 Medical University of Vienna, Vienna, Austria; 3 Clinic Hietzing, Vienna, Austria; 4 State Hospital Wiener Neustadt, Wiener Neustadt, Austria; 5 University Hospital of St. Poelten, Karl Landsteiner University of Health Sciences, Krems, Austria; 6 Hospital of Wels-Grieskirchen, Wels, Austria; 7 Medical University of Innsbruck, Innsbruck, Austria; 8 State Hospital Klagenfurt, Klagenfurt, Austria; 9 Hospital of Elisabethinen, Linz, Austria; 10 Federal Academic Teaching Hospital, Feldkirch, Austria; 11 Institute for Medical Informatics, Statistics and Documentation, Medical University of Graz, Graz, Austria

**Keywords:** Psoriasis, Skin diseases

## Abstract

With the introduction of the latest class of biologic drugs targeting interleukin (IL)-23p19, three new, highly effective drugs can be used for the treatment of chronic plaque psoriasis. However, poorer skin improvement as well as higher rates of serious adverse events have been reported for patients under real-world conditions (outside clinical trials). This accounts especially for patients who have already been treated with biologic drugs. We therefore aimed to determine effectiveness and safety of IL-23p19 inhibitors in real-world patients by analysing data from the Psoriasis Registry Austria (PsoRA) in this observational, retrospective, multicentre cohort study. Data for 197 patients (52.3% biologic-non-naïve), who were treated with anti-IL-23p19 antibodies (127 guselkumab, 55 risankizumab and 15 tildrakizumab) for at least 3 months, were eligible for analysis. In general, biologic-non-naïve patients displayed a less favourable response to anti-IL-23 treatment as compared to biologic-naïve patients. However, after correction for previous biologic exposure, few differences in PASI improvement were detected among biologic-naïve and -non-naïve patients treated with different IL-23p19 inhibitors. This indicates that treatment effectiveness is not related to the class of the previously administered therapy in biologic-non-naïve patients. Therefore, IL-23p19 inhibitors represent a promising treatment alternative for patients who have not responded to previous biologics. However, as with other biologic agents (including IL-17 inhibitors), we did not observe an entirely satisfactory treatment response (i.e. PASI < 3 and/or PASI 75) to anti-IL-23 treatment in one out of four to five patients. Adverse events (mainly non-severe infections) were observed in 23 (11.7%) patients with no major differences regarding the administered IL-23 inhibitor or previous biologic exposure.

## Introduction

With the introduction of the latest class of biologic drugs targeting interleukin (IL)-23p19, three new, highly effective drugs, i.e. guselkumab, risankizumab and tildrakizumab, can be used for the treatment of chronic plaque psoriasis^1–3^. However, 14.6–58.6% of patients with psoriasis treated in daily routine would not have been eligible for clinical trials; indeed, poorer skin improvement as well as higher rates of serious adverse events have been reported for those patients under real-world conditions^4,5^. Previous biologic exposure is a well-known risk factor for decreased drug survival, and it appears that clinical effectiveness is also reduced in these patients, including patients treated with IL-17 and IL-23 inhibitors^6–10^. These considerations are especially pertinent in order to select a new treatment, if treatment has been discontinued due to insufficient skin improvement, whereas the occurrence of adverse events often leads to a switch in biologic class or conventional systemic treatment^11,12^. Thus, recently developed non-invasive tools which might help predict the patients’ responses to biologic treatment could become a milestone in anti-psoriatic treatment^12^. Clinical trials of these tools are ongoing, and they will hopefully soon enable physicians to more efficiently select the most promising biologic drug for biologic-naïve and -non-naïve patients. Nevertheless, treatment selection depends on a variety of factors including disease severity, involvement of sensitive body sites, quality of life, response to previous therapies, comorbidities (including chronic infections), patient`s scheme of life, (and physician’s) and patient’s treatment preferences^13,14^. Thus, psoriasis patients need an individually tailored treatment selection^13,14^.

Recent case series have shown that intra-class switching within the group of IL-17 or IL-23 inhibitors can be a promising therapeutic option in patients exhibiting treatment failure^15,16^. However, little is known at this time about the impact of previous biologic exposure on subsequent treatments, not allowing physicians to draw conclusions regarding the advisability of intra- or inter-class biologic treatment switching^15,16^. Therefore, we evaluated both the treatment effectiveness in patients treated with guselkumab, risankizumab, or tildrakizumab and the influence of previous biologic exposure in these patients.

## Methods

### Study design

This study was carried out as an observational retrospective multicentre analysis of clinical data extracted from the Psoriasis Registry Austria (PsoRA). The design of this registry has been described in previous studies^7,17–19^. Further information about PsoRA and participating centres is available at www.psoriasisregistry.at. In the registry, one treatment is defined as the time from a patient`s allocation to a specific therapy, followed by at least one visit, until last observation or discontinuation of treatment. For every visit entered into the registry, the continuous prescription of a drug has to be confirmed; otherwise, the reason for treatment discontinuation has to be entered. The registry has been approved by the Ethics Committee of the Medical University of Graz (application number 21-094 ex 09/10), and the present analysis was conducted in accordance with the principles of the Declaration of Helsinki and informed consent has been obtained from the patients according to prerequisites of the study approval.

### Data analysis and statistics

The study population included patients > 18 years of age who had chronic plaque psoriasis and started a biologic therapy with guselkumab, risankizumab, or tildrakizumab. These patients had at least one follow-up visit (and a treatment duration of at least 3 months) with the same treatment, irrespective of their previous systemic treatment, psoriatic arthritis, or any comorbidities. Data extraction was performed on February 4, 2021, and covered the period from March 23, 2018, to February 4, 2021. The Psoriasis Area and Severity Index (PASI) score prior to therapy start and at least one PASI score during the follow-up (at 3, 6, or 12 months) had to be documented in order to include a patient in the analysis. The effectiveness of the IL-23 inhibitors was evaluated in terms of absolute PASI change and PASI reduction (defined as categories ranging from complete remission, i.e. PASI 100, to partial remission, i.e. PASI 90, PASI 75, PASI 50, PASI < 50 to worsening) with regard to biologic naivety, as well as in terms of the class of previous biologic therapy (i.e. tumour necrosis factor-TNF-alpha, IL-12/23, IL-23 and IL-17 inhibitors). The change in PASI was calculated and analysed as observed and with respect to the last observation carried forward (LOCF) worst-case scenario (considering the last known PASI or PASI reduction response to be continued or otherwise patients to be non-responders, i.e. PASI < 50 response) for further analysis, irrespective of treatment discontinuation.

The chi-square test was used to determine the treatment allocation concerning gender, psoriatic arthritis, and biologic naivety, as well as to analyse differences in the achievement of PASI reduction categories and to detect the occurrence of adverse events regarding the (class of) previous biologic exposure. Two sample *t*-tests and analysis of variances or the Kruskal–Wallis test were used to compare the PASI regarding the drug and (class of) previous biologic exposure. A post hoc analysis was performed pairwise using the Bonferroni correction. Calculations were performed with IBM® SPSS® Statistics 26.0 (Armonk, New York, IBM Corporation). *P*-values < 0.05 were considered to be statistically significant.

## Results

### General patient characteristics

Nine patients were excluded from the analysis due to treatment discontinuation prior to 3 months, and only two of these patients received more than one treatment dosage. Reasons for treatment discontinuation included patient requests due to lack of skin improvement (*n* = 4), side effects (*n* = 1) (skin abscess) and worsening of another skin disease (*acne inversa*) (*n* = 1), and other reasons (*n* = 3). PASI or PASI reduction was not available for these 9 patients. Data from 197 patients (34.0% women) and their administered treatments (127 cycles of guselkumab, 55 of risankizumab and 15 cycles of tildrakizumab) were eligible for analysis (Table 1). Psoriatic arthritis was present in 51 (25.9%) patients, and 94 (47.7%) of the treatments were administered in biologic-naïve patients (Table 1). In biologic-non-naïve patients, Il-17 and Il-12/23 inhibitors were the most frequently administered drugs (Table 1). Furthermore, 12.7% of patients had already received at least three (3) biologic treatments. No differences existed in allocation to treatment with regard to gender, age, BMI, PASI, biologic naivety, or class of previous biologic therapy (Table 1). All patients received standard dosage at treatment start. An off-label dosage change was performed in one man receiving risankizumab (in whom the risankizumab administration interval was reduced to 10 weeks) and in another man receiving tildrakizumab (in whom the dosage was increased to 200 mg tildrakizumab every 12 weeks). Fifty-two patients (26.4%) had a PASI ≤ 3 at treatment start (Table 1). Concomitant psoriatic arthritis was present in 24 of these patients (46.2%), and 35 patients (67.3%) were switched from another biologic to anti IL-23 treatment for various reasons (data not shown). Forty-eight (92.3%) patients suffered from the involvement of at least one difficult-to-treat or psychologically incriminating body site: 21 (40.4%) scalp, 17 (32.7%) nails, 14 (26.9%) inverse, 11 (21.2%) palmar and/or plantar region; 2 (3.8%) patients had additional palmoplantar involvement with pustules and plaques. Previous biologic treatment had been discontinued due to primary treatment failure (*n* = 10), secondary treatment failure (i.e. loss of efficacy) (*n* = 15), side effects (*n* = 4), patient request (*n* = 3), denial of reimbursement (*n* = 1), start of the COVID-19 pandemic (*n* = 1) and unknown reasons (*n* = 1) (data not shown).

**Table 1:** Patient characteristics.

Treatment characteristics		Treatment				*p*-value
		Guselkumab	Risankizumab	Tildrakizumab	All treatments	
Total number of treatments/patients		127	55	15	197	
Characteristic at start of treatment	Number (%) of females	44 (34.6)	19 (34.5)	4 (26.7)	67 (34.0)	0.891
	Mean age (SD)	46.0 (14.2)	47.9 (11.6)	45.9 (14.1)	46.5 (13.5)	0.668
	Mean PASI (SD) in biologic naïve patients	9.63 (6.59)	11.13 (6.67)	11.67 (11.08)	10.25 (7.23)	0.545
	Mean PASI (SD) in biologic non-naïve patients	7.80 (7.52)	9.13 (8.10)	9.13 (5.22)	8.01 (7.44)	0.700
	Number (%) of patients with PASI ≤ 3	37 (29.1)	12 (21.8)	3 (20.0)	52 (26.4)	0.496812
	Number (%) of cycles in patients with arthritis	31 (24.4)	19 (34.5)	1 (6.7)	51 (25.9)	0.077
	Mean weight in kg (SD)	92.0 (19.2)	88.9 (13.4)	94.4 (11.8)	91.3 (17.4)	0.736
	Mean BMI (SD)	30.0 (6.5)	29.3 (4.4)	29.9 (2.3)	29.8 (5.6)	0.914
Number (%) of patient with the last previous biologic treatment	None	54 (42.5)	29 (52.7)	11 (73.3)	94 (47.7)	0.068
	TNF-alpha	14 (11.0)	6 (10.9)	0 (0)	20 (10.2)+	
	IL-12/23	33 (26.0)	5 (9.1)	2 (13.3)	40 (20.3)*	
	IL-17	26 (20.5)	15 (27.3)	2 (13.3)	43 (21.8)#	
Number (%) of previous biologic therapies	1	41 (55.4)	13 (50.0)	2 (50.0)	56 (53.8)	0.767
	2	14 (18.9)	8 (30.8)	1 (25.0)	23 (22.1)	
	≥ 3	19 (25.7)	5 (19.2)	1 (25.0)	25 (24.0)	

Analysis of variances and chi-square test did not reveal differences in allocation to treatment regarding sex, age, PASI, psoriatic arthritis, or (type of) previous biologic exposure. Chi-square test results revealed that a previous treatment with TNF-alpha and IL-12/23 inhibitors were the first biologic treatments for a significantly higher proportion of patients (*p* = 0.000778).

+Seven patients (35.0%) were biologic-non-naïve when treated with TNF-alpha inhibitors.

*Ten patients (25.0%) were biologic-non-naïve when treated with IL-12/23 inhibitors.

#Twenty-eight patients (65.1%) were biologic-non-naïve when treated with IL-17 inhibitors.

BMI, body mass index; IL, interleukin; PASI, Psoriasis Area and Severity Index; SD, standard deviation; TNF, tumour necrosis factor.

### Effectiveness

**Figure 1:**
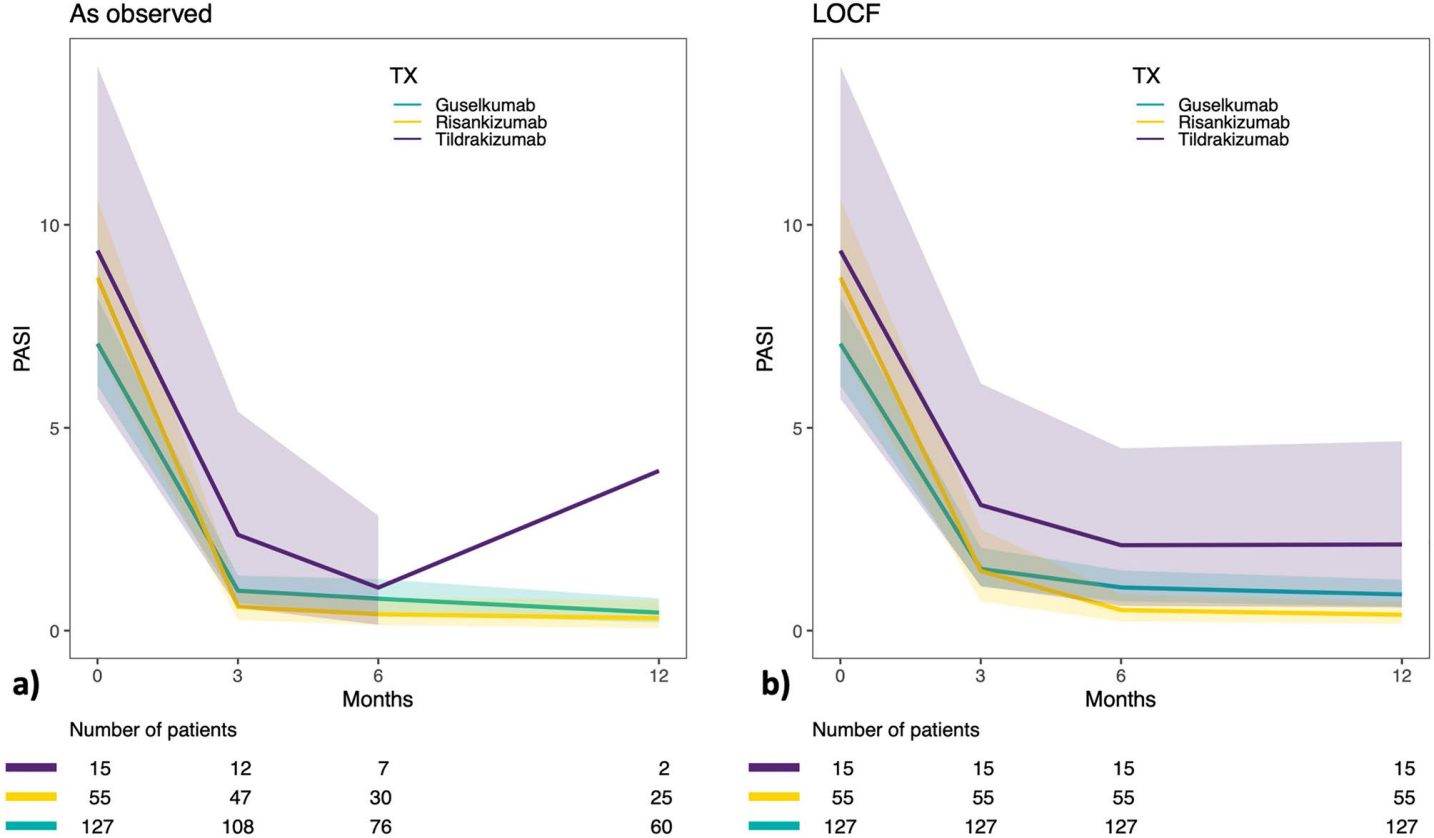
Effectiveness of IL-23 inhibitors in terms of absolute PASI. Absolute Psoriasis Area and Severity Index (PASI) value (± 95% confidence interval) plotted over time for patients analysed as observed (**a**) and per last observation carried forward (LOCF) (**b**).

The mean (SD) PASI at treatment start was 8.42 (7.13), 10.07 (7.28) and 11.0 (9.74) for patients treated with guselkumab, risankizumab and tildrakizumab, respectively (Table S1). PASI (SD) continuously declined over 12 months to 1.22 (2.84)/1.93 (3.64) for guselkumab, 0.93 (1.80)/1.03 (1.94) for risankizumab and 5.40 (6.79)/3.93 (6.57) for tildrakizumab regarding the analysis as observed/LOCF (Fig. 1, Table S1). In general, biologic-naïve patients had a significantly higher disease severity at treatment start with a mean PASI (SD) of 10.25 (7.23) as compared to 8.01 (7.44) in biologic-non-naïve patients (*p* = 0.033) (Table 2). However, biologic-naïve patients achieved significantly lower mean absolute PASI values (SD) as compared to -non-naïve patients at 3 months (1.27 [3.01] versus 2.35 [3.83]; *p* = 0.048) and 6 months (0.53 [0.82] versus 2.86 [4.45]; *p* = 0.001). Furthermore, a trend toward lower PASI at 12 months was detected in biologic-naïve patients as compared to -non-naïve patients (*p* = 0.07) (Table 2). However, PASI values were similar for each timepoint within the groups of biologic-naïve and -non-naïve patients, irrespective of the currently administered Il-23 inhibitor, except for PASI values at 12 months in biologic-non-naïve patients (*p* = 0.006) (Table 3). The class of the previously administered biologic drug had no significant influence on PASI effectiveness in patients treated with IL-23p19 inhibitors at 3 months (*p* = 0.312), 6 months (*p* = 0.535), or 12 months (*p* = 0.999) after treatment initiation, despite the significant differences observed in PASI at treatment start regarding class of previous biologic treatment (*p* = 0.001) (Table 4).

**Table 2:** Treatment effectiveness regarding biologic naivety.

Timepoint	Mean PASI (SD) in biologic-naïve/-non-naïve patients as observed		*p*-value
	Biologic naive	Biologic non-naive	
Baseline	10.25 (7.23)	8.01 (7.44)	**0.033**
3 months	1.27 (3.01)	2.35 (3.83)	**0.048**
6 months	0.53 (0.82)	2.86 (4.45)	** < 0.001**
12 months	0.71 (2.68)	1.77 (2.69)	0.07

A two-sample *t*-test revealed significantly higher PASI values in biologic naïve patients at treatment start, as well as significantly higher PASI values in biologic-non-naïve patients at 3 and 6 months after treatment initiation.

Significant *p*-values are in bold. PASI, Psoriasis Area and Severity Index; SD, standard deviation.

**Table 3:** Treatment effectiveness as observed regarding biologic naivety in patients treated with different IL-23 inhibitors.

Timepoint	Treatment	Biologic naïve patients		Biologic-non-naïve patients	
		PASI (SD)	*p*-value	PASI (SD)	*p*-value
Baseline	Guselkumab	9.63 (6.59)	0.545	7.80 (7.52)	0.700
	Risankizumab	11.13 (6.67)		9.13 (8.10)	
	Tildrakizumab	11.67 (11.08)		9.13 (5.22)	
3 months	Guselkumab	1.02 (1.55)	0.085	2.59 (4.44)	0.200
	Risankizumab	0.95 (1.59)		2.08 (2.76)	
	Tildrakizumab	3.56 (7.78)		6.47 (3.56)	
6 months	Guselkumab	0.54 (0.86)	0.745	3.25 (5.07)	0.798
	Risankizumab	0.42 (0.75)		2.48 (3.22)	
	Tildrakizumab	0.74 (0.86)		4.20 (2.55)	
12 months	Guselkumab	1.01 (3.34)	0.609	1.42 (2.41)	**0.006**
	Risankizumab	0.107 (0.29)		2.76 (3.29)	
	Tildrakizumab	0.60 (NA)		10.20 (NA)	

Analysis of variances revealed no statistically significant differences in PASI for any timepoint among biologic-naïve or -non-naïve patients with regard to the administered drug, except for PASI at 12 months in biologic-non-naïve patients. However, post hoc analysis was not feasible due to the low number of patients at 12 months in the tildrakizumab group (*n* = 2).

Significant *p*-values are bold.

NA, not applicable (none); PASI, Psoriasis Area and Severity Index; SD, standard deviation.

**Table 4:** Treatment effectiveness as observed with regard to class of previous biologic therapy.

Timepoint (total number of patients)	Type of biologic	Number (percentage) of patients that received a certain class of previous biologic therapy	Biologic-non-naïve patients	
			PASI (SD)	*p*-value
Baseline (103)	TNF-α	20 (19.4)	7.36 (5.52)	**0.001**
	Il-17	43 (41.7)	11.12 (8.94)	
	Il-12/23	40 (38.8)	4.99 (4.86)	
3 months (91)	TNF-α	17 (18.7)	2.17 (3.29)	0.312
	Il-17	34 (37.4)	3.12 (5.23)	
	Il-12/23	38 (41.6)	1.74 (2.24)	
6 months (55)	TNF-α	10 (18.2)	1.56 (2.32)	0.535
	Il-17	28 (50.9)	3.40 (5.05)	
	Il-12/23	17 (30.9)	2.72 (4.40)	
12 months (43)	TNF-α	5 (11.6)	1.80 (2.00)	0.999
	Il-17	19 (44.2)	1.78 (2.89)	
	Il-12/23	19 (44.2)	1.75 (2.78)	

Analysis of variances revealed statistically significant differences in disease severity (as measured in PASI) regarding the class of previous biologic treatment at baseline. Post hoc analysis results show significantly higher PASI in patients treated with IL-17 inhibitors as compared to those treated with IL-23 inhibitors (*p* < 0.001), but not TNF- α inhibitors (*p* = 0.147). There was no difference regarding PASI at baseline between TNF- α inhibitors and IL-12/23 inhibitors at baseline (*p* = 0.655).

Significant *p*-values are in bold.

IL, interleukin; PASI, Psoriasis Area and Severity Index; SD, standard deviation; TNF, tumour necrosis factor.

**Figure 2:**
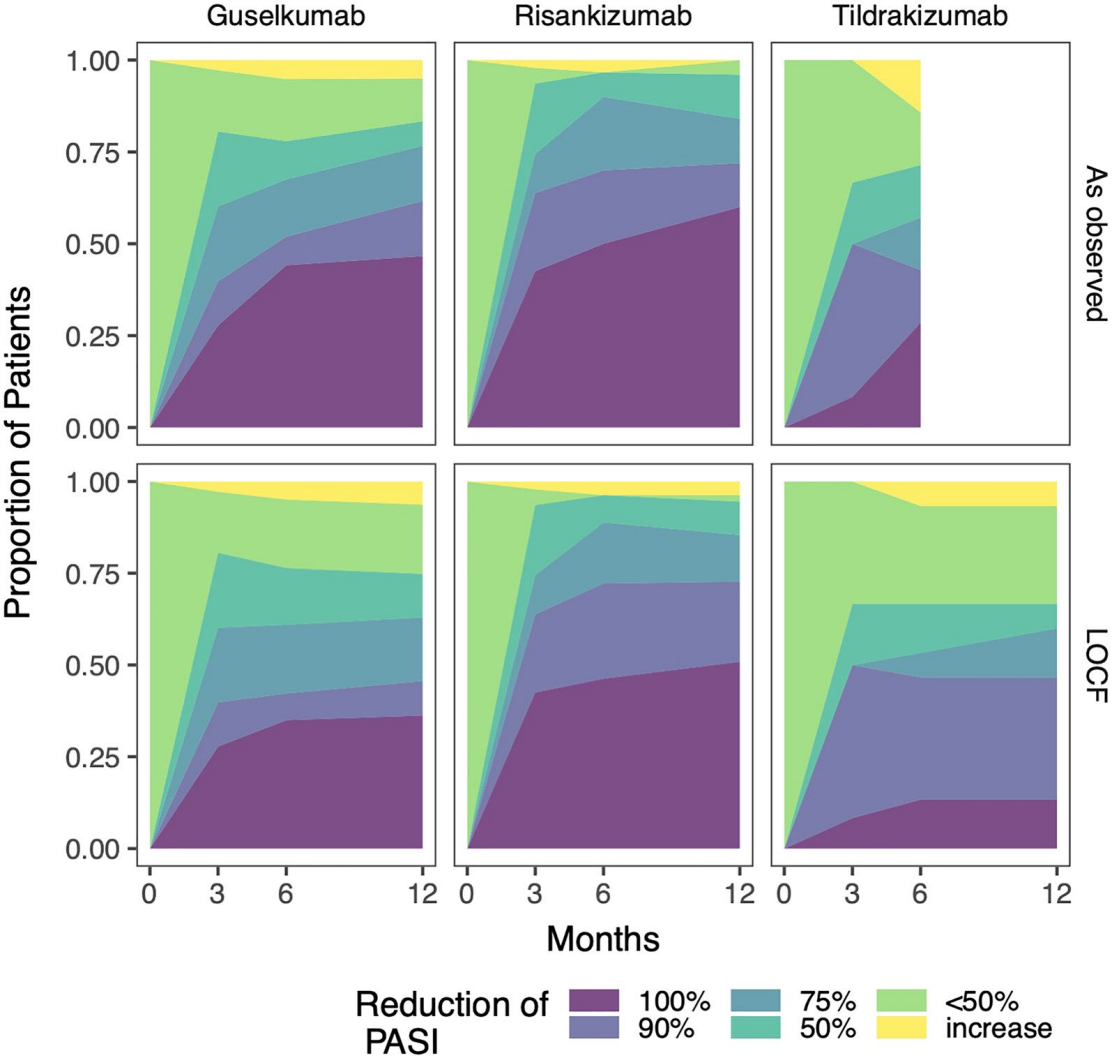
Achievement of skin goals. Relative number of patients analysed as observed and per last observation carried forward (LOCF), in whom a certain Psoriasis Area and Severity Index (PASI) was achieved, plotted over time.

After 3 months of treatment, the observed PASI 50, 75, 90 and 100 (complete response) rates were 80.6%, 60.2%, 39.8% and 27.8% for guselkumab, 93.6%, 74.5%, 63.9% and 42.6% for risankizumab, and 66.7%, 50.0%, 50.0% and 8.3% for tildrakizumab, respectively. These values ultimately reached 83.4%, 76.7%, 61.7% and 46.7% for guselkumab, 96.0%, 84.0%, 72.0% and 60.0% for risankizumab and 50.0%, 50.0%, none (not available) and none (not available) for tildrakizumab after 12 months of treatment (Fig. 2, Table 5). In general, a significantly higher proportion of biologic-naïve patients achieved PASI 75, PASI 90 and PASI 100 responses at 3 (*p* = 0.0049), 6 (*p* = 0.0041) and 12 months (*p* = 0.0413) after treatment initiation compared to non-naïve patients. However, these findings could only be statistically confirmed in the subgroup analysis for risankizumab at 6 months (*p* = 0.0001), and for tildrakizumab at 3 months (*p* = 0.0013) after treatment initiation (Table S2).

**Table 5:** Achievement of treatment goals.

Treatment	Timepoint (months)	Number of patients (as observed/LOCF)	Percentage of patients achieving a certain PASI reduction (as observed/LOCF)						Number (%) of patients of patients achieving ≤ PASI 3 (as observed /LOCF)
			PASI 100	> PASI 90	> PASI 75	> PASI 50	< PASI 50	Increase in PASI	
Guselkumab	3	108/127	27.8/23.6	39.8/33.8	60.2/51.1	80.6/68.4	16.7/29.1	2.8/2.4	89 (82.4)/94 (74.0)
	6	77/127	44.2/33.9	52.0/41.0	67.6/59.1	78.0/74.1	16.9/21.3	5.2/4.7	65 (84.4)/104 (81.9)
	12	60/127	46.7/36.2	61.7/45.6	76.7/62.9	83.4/74.7	11.7/18.9	5.0/6.3	53 (88.3)/105 (82.7)
Risankizumab	3	47/55	42.6/36.4	63.9/54.6	74.5/63.7	93.6/80.1	4.3/18.2	2.1/1.8	41 (87.2)/41 (74.5)
	6	30/55	50.0/45.5	70.0/71.0	90.0/87.4	96.7/94.7	NA/1.8	3.3/3.6	27 (90.0)/48 (87.3)
	12	25/55	60.0/50.9	72.0/72.7	84.0/85.4	96.0/94.5	4.0/1.8	NA/3.6	22 (88.0)/48 (87.3)
Tildrakizumab	3	12/15	8.3/6.7	50.0/40.0	50.0/40.0	66.7/53.3	33.3/46.7	NA/NA	9 (75.0)/10 (66.7)
	6	7/15	28.6/13.3	42.9/46.6	57.2/53.3	71.5/66.6	14.3/26.7	14.3/6.7	6 (85.7)/11 (73.3)
	12	2/15	NA/13.3	NA/46.6	50.0/59.9	50.0/66.6	NA/26.7	50.0/6.7	1 (50.0)/11 (73.3)
All treatments (*N* = 197)	3	167/197	29.9/25.4	47.3/40.1	63.5/53.8	82.0/70.6	14.4/27.4	2.4/2.0	139 (83.2%)/145 (73.6%)
	6	114/197	44.7/35.5	56.1/49.7	72.8/66.5	82.5/79.2	12.3/16.2	5.3/4.6	98 (86.7%)/163 (82.7%)
	12	87/197	49.4/38.6	63.2/53.3	78.2/69.0	86.2/79.7	9.2/14.7	4.6/5.6	76 (87.4%)/164 (83.3%)
Patients with PASI > 3 at treatment start (*n* = 145)	3	131/145	30.5/27.6	51.1/46.2	64.1/57.9	80.9/73.1	9.9/25.5	1.5/1.4	94 (77.7%)/94 (64.8%)
	6	86/145	50.0/37.9	62.8/55.2	81.4/48.3	91.9/85.5	5.8/11.7	2.3/2.8	75 (87.2%)/116 (80.0%)
	12	63/145	55.6/41.4	71.4/59.3	85.7/76.6	93.7/86.9	4.8/9.7	1.6/3.4	54 (85.7)/116 (80.0%)

In 52 patients, the PASI at baseline was ≤ 3, of whom 9 had a PASI ≤ 1 at baseline. The number (%) of those patients achieving a PASI ≤ 1 as observed/LOCF was 32 (69.6)/32 (61.5), 16 (59.3)/34 (65.4), 18 (75.0)/35 (67.3) at 3, 6 and 12 months, respectively. In 2/4 patients, the PASI increased to > 3 at 12 months with regard to the analysis as observed/LOCF, while the remaining patients had a stable disease (continued having PASI ≤ 3).

LOCF, last observation carried forward; NA, not applicable (none); PASI, Psoriasis Area and Severity Index.

### Safety

Adverse events were documented in 23 patients (11.7%), with infections being the most commonly seen side effects in 18 patients (9.1%), taking all IL-23 inhibitors together (Table 6). No differences were identified in the incidence of adverse events with regard to the administered drug (*p* = 0.159) (Table S3). Furthermore, the rates of adverse events were similar in biologic-naïve and -non-naïve patients (*p* = 0.368294) (Table 6).

**Table 6:** Occurrence of adverse events under treatment with Il-23 inhibitors.

Type of adverse event	Number (%) of patients reporting an adverse event once per drug			
	All (*N* = 197)	Guselkumab (*n* = 127)	Risankizumab (*n* = 55)	Tildrakizumab (*n* = 15)
Gastrointestinal symptoms	2 (1.02)+	1 (0.79)	1 (1.82)	NA
Infection	18 (9.14)*	16 (12.60)	2 (3.64)	NA
Neurological symptoms	2 (1.02)#	1 (0.79)	NA	1 (6.67)
Rash	1 (0.05)	1 (0.79)	NA	NA
All	23 (11.67)	19 (14.96)	3 (5.45)	1 (6.67)

Fisher’s exact test results indicate no significant differences in the occurrence of adverse events regarding drug (*p* = 0.159). *N* = 197.

NA, not applicable (none).

+Included two patients with diarrhoea.

*Included seven patients with common cold, two patients with coronavirus disease 2019 (COVID-19), two patients with cellulitis, one patient with influenza, one patient with herpes zoster, one patient with skin abscess, one patient with angina tonsillaris, one patient with tooth abscess, and two patients with unspecified infections.

#Included one patient with headache and one patient with headache and paraesthesia.

### Treatment discontinuation

A total of 22 (11.2%) out of 197 patients discontinued treatment within the first treatment year (guselkumab, 12.6%; risankizumab, 5.5%; tildrakizumab, 13.3%). Reasons for treatment discontinuation included primary treatment failure (*n* = 7, 3.6%), secondary treatment failure (i.e. loss of efficacy) (*n* = 5, 2.5%), insecurities due to the COVID-19 pandemic (n = 5, 2.5%), side effects (*n* = 3, 1.5%) and patient request (*n* = 2, 1.0%).

## Discussion

This study of 197 patients is one of the larger registry studies conducted so far to examine the effectiveness and safety of real-world patients receiving IL-23p19 inhibitors, as well as the impact of previous biologic exposure on these parameters. The rate of off-label dosage changes were relatively low, i.e. 1.0% in this study as compared to that of 14.1% for the IL-12/23 inhibitor ustekinumab, which was been reported from this registry previously^7^. Taking all anti-IL-23 inhibitors together, the observed/LOCF response rates at 3 months with a PASI 100 were 29.9%/25.4%; for PASI 90, 47.3%/40.1%; and for PASI 75, 63.5%/53.8%. After 12 months, the values for PASI 100 reached 49.4%/38.6%; for PASI 90, 63.2%/53.3%; and for PASI 75, 78.2%/69.0%.

Treatment effectiveness in terms of the PASI reduction category for guselkumab observed in this study was within the lower range or was slightly lower than that reported in clinical trials. Three months after treatment start, the PASI 75 response was 60.2% (as compared to 69.8–91.2%), the PASI 90 response was 39.8% (as compared to 69.0–73.3%), and the PASI 100 response was 27.8% (as compared to 27.0–37.4%). Fifty-two weeks after treatment start, the PASI 75 response was 76.7% (as compared to 77.8–87.8%), the PASI 90 response was 61.7% (as compared to 76.3–84.0%), and the PASI 100 response was 46.7% (as compared to 46.7–58.0%) (Fig. 2, Table 5)^20–22^. In general, these response rates are also in the range (or slightly lower) as those recently published from Italian real-world patient cohorts^23–25^.

Treatment results for risankizumab were also within the lower range or slightly lower than results from a clinical trial. PASI 75 responses at 12 weeks were 74.5% (as compared to 91.0–94.5%), PASI 90 responses were 63.9% (as compared to 72.0–74.8%), and PASI 100 responses were 42.6% (as compared to 32.7–40.0%). Fifty-two weeks after treatment start, the PASI 75 response was 84.0% (as compared to up to 96.4%), the PASI 90 response was 72.0% (as compared to 80.6–92.7), and the PASI 100 response was 60.0% (as compared to 41.8–60.0%) (Fig. 2, Table 5)^1,26^. Similar data regarding treatment effectiveness has been obtained in recently published real-world studies^27,28^.

However, it is noteworthy that the efficacy endpoints in most of these clinical trials (of guselkumab and risankizumab) were measured 16 weeks after treatment initiation, whereas this study analysed treatment effectiveness 12 weeks after treatment initiation^1,21,22,26,29^. Furthermore, the rate of biologic-non-naïve patients in this study was higher than that in clinical trials, namely, 57.5% and 47.3% biologic-non-naïve patients for guselkumab and risankizumab, respectively, as compared to a range of 17.5–29.0% in guselkumab trials and 29.0–39.0% in risankizumab trials (Table 1)^1,20–22,26^.

Patients receiving tildrakizumab had treatment responses that mostly fell well within the range of results cited from clinical trials, with a PASI 75 response 12 weeks in this study of 50% (as compared to 41.2–64.0%), a PASI 90 response of 50.0% (as compared to 35.0–73.2%), and a PASI 100 response of 8.3% (as compared to 14.0–34.4%). Meanwhile, twenty-six weeks after treatment initiation, a PASI 75 response of 57.2% was recorded (as compared to 73.0–80.0%), a PASI 90 response of 42.9% (as compared to 52.0–56.0%), and a PASI 100 response of 28.6% (as compared to 23.0–24.0%) (Fig. 2, Table 5)^30–32^. However, better reals-word response rates have been recently reported in Italian patient cohorts^25,33^. Like the rates for biologic-non-naïve patients seen in the clinical trials of guselkumab and risankizumab, the rates seen for biologic-non-naïve patients receiving tildrakizumab were 26.7% in this study; thus, this rate was slightly higher than the rate seen in its clinical trials (13.0–23.0%) (Table 1). However, the PASI < 50 response rates at 6/12 months after treatment initiation were relatively high in patients treated with guselkumab (21.3%/18.9%) and tildrakizumab (26.7% and 26.7%, respectively) as compared to risankizumab (1.8%/1.8%) in the LOCF analysis. Significantly better skin improvement as measured by absolute PASI was also observed at 12 months for risankizumab as compared to tildrakizumab (Table S1). It is important to note, however, that PASI reduction is not the most appropriate method to measure treatment effectiveness in patients with low PASI at baseline^34^. Notably, 52 patients (26.4%) included in this study started treatment with PASI ≤ 3 (Table 1). After excluding these patients, the LOCF analysis results reveal that 80.0% of the patients achieved a PASI ≤ 3 at 12 months, which is considered as achieving the therapeutic treatment goal (Table 5)^35^. On the other hand, this finding indicates that 1 out of 5 patients did not display an entirely satisfactory treatment response (Table 5). With regard to PASI reduction, 23.4% did not reach a PASI 75 response, and 13.1% (i.e. one out of eight patients) remained below a PASI 50 improvement level or their skin manifestations even worsened (Table 5). Notably, similar PASI reduction rates were observed when patients starting with PASI ≤ 3 were not excluded from analysis (Table 5).

Analysing the treatment outcomes of the whole cohort with regard to previous biologic exposure revealed higher rates of PASI 50, PASI 75, PASI 90 and PASI 100 responses in biologic-naïve patients as compared to in biologic-non-naïve patients at 3, 6 and 12 months after treatment initiation (Table S2). These findings are consistent with results from clinical trials and first real-world data, which show a better treatment outcome in biologic-naïve patients or patients directly randomized to the IL-23p19 arm instead of the biologic crossover arm^9,23,26,30,32,36,37^. However, it appears as though the class of the previously administered biologic does not influence the effectiveness of the IL-23p19 inhibitors (Table 4). It is noteworthy, that patients previously treated with IL-17 inhibitors had the highest PASI values at baseline among the biologic-non-naïve patients (Table 4). This may indicate a more difficult-to-treat patient cohort, as significantly more patients had already received more than one previous biologic treatment in the IL-17 group (65.1%) as compared to those in the TNF-alpha (35.0%) and IL12/23 (25.0%) groups (Table 1). This finding is consistent with those of another analysis of data from the Austrian registry, whereby patients treated with IL-17 inhibitors were significantly more often biologic-non-naïve patients^7^. The findings indicate that switching from IL-17 inhibitors to IL-23 inhibitors might be a promising therapeutic alternative if treatment with IL-17 inhibitors fails. Nevertheless, biologic-naïve patients showed a significantly higher PASI improvement for all measured timepoints, despite having a higher skin burden at treatment start (Table 2). However, after correcting for previous biologic exposure, no major differences in PASI improvement could be detected in PASI for any timepoint among biologic-naïve or -non-naïve patients with regard to the administered drug, except for PASI at 12 months in biologic-non-naïve patients. (Table 3). These findings indicate that all IL-23 inhibitors are promising drugs for the subsequent treatment of biologic-non-naïve patients.

In general, the rate of adverse events (11.67%) observed in this study is much lower than that reported in clinical trials, as well as real-world patients (Table 6)^20,24,28,29,31^. The rates of adverse events found in this study were also similar to previously published rates between biologic-naïve and non-naïve patients (Table S3)^38^.

### Limitations

The registry`s retrospective design aside, the limitations of this study include the fact that only a low number of patients received tildrakizumab, limiting the validity of results reported for this drug. Furthermore, more patients than usual could have deviated from the regular administration of the prescribed drug due to the ongoing pandemic for several reasons (e.g. being placed under quarantine, waiting for vaccination, or worrying about developing a more severe course of COVID-19 while/due to taking immunomodulatory drugs).

## Conclusion

IL-23 inhibitors are highly effective drugs for the treatment of chronic plaque psoriasis with biologic-naïve patients, enabling them to achieve better skin improvement than biologic-non-naïve patients. In biologic-non-naïve patients, the treatment effectiveness is not related to the class of the previously administered therapy. Therefore, IL-23p19 inhibitors represent a promising treatment alternative in patients for whom previous biologic treatment has failed. However, despite all of the improvements and progress made in the treatment of chronic plaque type psoriasis as reported from clinical studies and the fact that IL-23 inhibitors (together with anti-IL-17 inhibitors) are considered as the most effective class of anti-psoriatic drugs, the study also revealed that one patient out of four to five treated with these drugs under real-world conditions (i.e. outside of clinical studies) still does not achieve an entirely satisfactory treatment response (i.e. PASI < 3 and/or PASI 75). This makes the continued improvement of anti-psoriatic drugs desirable in order to provide a satisfactory response to all patients in need.

### Supplementary Information

Supplementary Information.

## Data Availability

The datasets analysed during the current study are available from the corresponding author on reasonable request.

## References

[1] Gordon KB (2018). Efficacy and safety of risankizumab in moderate-to-severe plaque psoriasis (UltIMMa-1 and UltIMMa-2): results from two double-blind, randomised, placebo-controlled and ustekinumab-controlled phase 3 trials. Lancet.

[2] Reich K (2019). Maintenance of clinical response and consistent safety profile with up to three years of continuous treatment with guselkumab: results from the VOYAGE 1 and VOYAGE 2 trials. J. Am. Acad. Dermatol..

[3] Blauvelt A (2019). Tildrakizumab efficacy and impact on quality of life up to 52 weeks in patients with moderate-to-severe psoriasis: a pooled analysis of two randomized controlled trials. J. Eur. Acad. Dermatol. Venereol..

[4] Mason KJ (2018). Comparison of drug discontinuation, effectiveness, and safety between clinical trial eligible and ineligible patients in BADBIR. JAMA Dermatol..

[5] Masson Regnault M (2019). Users of biologics in clinical practice: would they be eligible for phase III clinical studies? Cohort study in the French Psoriasis Registry PSOBIOTEQ. J. Eur. Acad. Dermatol. Venereol..

[6] Mourad A, Straube S, Armijo-Olivo S, Gniadecki R (2019). Factors predicting persistence of biologic drugs in psoriasis: a systematic review and meta-analysis. Br. J. Dermatol..

[7] Graier T (2020). Biologic drug survival rates in the era of anti-Il-17 antibodies: a time period-adjusted registry analysis. Br. J. Dermatol..

[8] Papp KA (2018). Impact of previous biologic use on the efficacy and safety of brodalumab and ustekinumab in patients with moderate-to-severe plaque psoriasis: integrated analysis of the randomized controlled trials AMAGINE-2 and AMAGINE-3. Br. J. Dermatol..

[9] Strober B (2020). Efficacy of risankizumab in patients with moderate-to-severe plaque psoriasis by baseline demographics, disease characteristics and prior biologic therapy: an integrated analysis of the phase III UltIMMa-1 and UltIMMa-2 studies. J. Eur. Acad. Dermatol. Venereol..

[10] Mazzotta A, Esposito M, Costanzo A, Chimenti S (2009). Efficacy and safety of etanercept in psoriasis after switching from other treatments: an observational study. Am. J. Clin. Dermatol..

[11] Hu Y, Chen Z, Gong Y, Shi Y (2018). A review of switching biologic agents in the treatment of moderate-to-severe plaque psoriasis. Clin. Drug Investig..

[12] Strober B (2021). A Survey of Community dermatologists reveals the unnecessary impact of trial-and-error behavior on the psoriasis biologic treatment paradigm. Dermatol. Ther. (Heidelb).

[13] Kaushik SB, Lebwohl MG (2019). Psoriasis: Which therapy for which patient: Psoriasis comorbidities and preferred systemic agents. J. Am. Acad. Dermatol..

[14] Kaushik SB, Lebwohl MG (2019). Psoriasis: Which therapy for which patient: Focus on special populations and chronic infections. J. Am. Acad. Dermatol..

[15] Gasslitter I (2019). Successful intra-class switching among IL-17 antagonists: a multicentre, multinational, retrospective study. Arch. Dermatol. Res..

[16] Reddy R, Pannu S, Fiumara K, Kahn J, Rosmarin D (2021). Efficacy of in-class interleukin-23 inhibitor switching: risankizumab following guselkumab failure in moderate-to-severe psoriasis treatment. Br. J. Dermatol..

[17] Inzinger M (2014). Short- to intermediate-term follow-up in patients treated with the combination of 311-nm ultraviolet B phototherapy and biological agents. Br. J. Dermatol..

[18] Inzinger M (2016). Survival and effectiveness of tumour necrosis factor-alpha inhibitors in the treatment of plaque psoriasis under daily life conditions: report from the psoriasis registry Austria. Acta Derm. Venereol..

[19] Graier T, Weger W, Sator P, Salmhofer W (2020). Effectiveness and clinical predictors of drug survival in psoriasis patients receiving apremilast: a registry analysis. J. Am. Acad. Dermatol. Int..

[20] Reich K (2019). Guselkumab versus secukinumab for the treatment of moderate-to-severe psoriasis (ECLIPSE): results from a phase 3, randomised controlled trial. Lancet.

[21] Blauvelt A (2017). Efficacy and safety of guselkumab, an anti-interleukin-23 monoclonal antibody, compared with adalimumab for the continuous treatment of patients with moderate to severe psoriasis: results from the phase III, double-blinded, placebo- and active comparator. J. Am. Acad. Dermatol..

[22] Ohtsuki M (2018). Guselkumab, an anti-interleukin-23 monoclonal antibody, for the treatment of moderate to severe plaque-type psoriasis in Japanese patients: efficacy and safety results from a phase 3, randomized, double-blind, placebo-controlled study. J. Dermatol..

[23] Dapavo P (2021). Efficacy, safety, and drug survival of IL-23, IL-17, and TNF-alpha inhibitors for psoriasis treatment: a retrospective study. J. Dermatol. Treat..

[24] Megna M, Potestio L, Ruggiero A, Camela E, Fabbrocini G (2022). Guselkumab is efficacious and safe in psoriasis patients who failed anti-IL17: a 52-week real-life study. J. Dermatol. Treat..

[25] Megna M (2022). Real-world practice indirect comparison between guselkumab, risankizumab, and tildrakizumab: results from an Italian 28-week retrospective study. J. Dermatol. Treat..

[26] Reich K (2019). Risankizumab compared with adalimumab in patients with moderate-to-severe plaque psoriasis (IMMvent): a randomised, double-blind, active-comparator-controlled phase 3 trial. Lancet.

[27] Megna M, Potestio L, Ruggiero A, Camela E, Fabbrocini G (2022). Risankizumab treatment in psoriasis patients who failed anti-IL17: A 52-week real-life study. Dermatol. Ther..

[28] Ruggiero A (2022). Anti-interleukin-23 for psoriasis in elderly patients: guselkumab, risankizumab and tildrakizumab in real-world practice. Clin. Exp. Dermatol..

[29] Ohtsuki M (2019). Efficacy and safety of risankizumab in Japanese patients with moderate to severe plaque psoriasis: results from the SustaIMM phase 2/3 trial. J. Dermatol..

[30] Poulin Y (2020). Efficacy of tildrakizumab by patient demographic and disease characteristics across a phase 2b and 2 phase 3 trials in patients with moderate-to-severe chronic plaque psoriasis. J. Eur. Acad. Dermatol. Venereol..

[31] Reich K (2017). Tildrakizumab versus placebo or etanercept for chronic plaque psoriasis (reSURFACE 1 and reSURFACE 2): results from two randomised controlled, phase 3 trials. Lancet.

[32] Reich K (2020). Long-term efficacy and safety of tildrakizumab for moderate-to-severe psoriasis: pooled analyses of two randomized phase III clinical trials (reSURFACE 1 and reSURFACE 2) through 148 weeks. Br. J. Dermatol..

[33] Caldarola G (2022). Tildrakizumab in moderate-to-severe plaque psoriasis: A multicenter, retrospective, real-life study. Dermatol. Ther..

[34] Norlin JM, Nilsson K, Persson U, Schmitt-Egenolf M (2019). Complete skin clearance and Psoriasis Area and Severity Index response rates in clinical practice: predictors, health-related quality of life improvements and implications for treatment goals. Br. J. Dermatol..

[35] Carretero G (2018). Redefining the therapeutic objective in psoriatic patients candidates for biological therapy. J. Dermatol. Treat..

[36] Langley RG (2018). Efficacy and safety of guselkumab in patients with psoriasis who have an inadequate response to ustekinumab: results of the randomized, double-blind, phase III NAVIGATE trial. Br. J. Dermatol..

[37] Reich K (2017). Efficacy and safety of guselkumab, an anti-interleukin-23 monoclonal antibody, compared with adalimumab for the treatment of patients with moderate to severe psoriasis with randomized withdrawal and retreatment: results from the phase III, double-blind, p. J. Am. Acad. Dermatol..

[38] Gottlieb AB (2017). Treatment outcomes with ixekizumab in patients with moderate-to-severe psoriasis who have or have not received prior biological therapies: an integrated analysis of two Phase III randomized studies. J. Eur. Acad. Dermatol. Venereol..

