# Author Correction: Real-world effectiveness of anti-interleukin-23 antibodies in chronic plaque-type psoriasis of patients from the Austrian Psoriasis Registry (PsoRA)

**DOI:** 10.1038/s41598-024-57807-3

**Published:** 2024-03-26

**Authors:** T. Graier, W. Weger, C. Jonak, P. Sator, C. Zikeli, K. Prillinger, C. Sassmann, B. Gruber, W. Saxinger, G. Ratzinger, C. Painsi, A. Mlynek, N. Häring, B. Sadoghi, H. Trattner, R. Müllegger, F. Quehenberger, W. Salmhofer, Peter Wolf

**Affiliations:** 1 Department of Dermatology and Venereology, Medical University of Graz, Auenbruggerplatz 8, 8036 Graz, Austria; 2 Medical University of Vienna, Vienna, Austria; 3 Clinic Hietzing, Vienna, Austria; 4 State Hospital Wiener Neustadt, Wiener Neustadt, Austria; 5 University Hospital of St. Poelten, Karl Landsteiner University of Health Sciences, Krems, Austria; 6 Hospital of Wels-Grieskirchen, Wels, Austria; 7 Medical University of Innsbruck, Innsbruck, Austria; 8 State Hospital Klagenfurt, Klagenfurt, Austria; 9 Hospital of Elisabethinen, Linz, Austria; 10 Federal Academic Teaching Hospital, Feldkirch, Austria; 11 Institute for Medical Informatics, Statistics and Documentation, Medical University of Graz, Graz, Austria

Correction to: *Scientific Reports* 10.1038/s41598-022-18790-9, published online 05 September 2022

In the original version of this Article F. Quehenberger was incorrectly affiliated with ‘Department of Dermatology and Venereology, Medical University of Graz, Auenbruggerplatz 8, 8036 Graz, Austria’. The correct affiliation for F. Quehenberger is listed below.

Institute for Medical Informatics, Statistics and Documentation, Medical University of Graz, Graz, Austria.

The original Article has been corrected.

