# Supplementary material for: Real-world effectiveness of anti-interleukin-23 antibodies in chronic plaque-type psoriasis of patients from the Austrian Psoriasis Registry (PsoRA)
Source: Sci Rep. 2022 Sep 5;12:15078. doi: 10.1038/s41598-022-18790-9 (PMC9442573; doi:10.1038/s41598-022-18790-9)
Supplement: Supplementary file 1 — Supplementary Information. [file 41598_2022_18790_MOESM1_ESM.docx]

**Supplementary information files**

Table S1: Treatment effectiveness

Table S2: Achievement of treatment goals regarding previous biologic exposure

Table S3: Occurrence of adverse events

| **Timepoint (months)** | **Mean PASI (SD)** | | | | | | ***p*-value (as observed)** | ***p*-value (LOCF)** |
| --- | --- | --- | --- | --- | --- | --- | --- | --- |
|  | **Guselkumab** | | **Risankizumab** | | **Tildrakizumab** | |  |  |
|  | **As observed** | **LOCF** | **As observed** | **LOCF** | **As observed** | **LOCF** |  |  |
| **0** | 8.42 (7.13) | | 10.07 (7.28) | | 11.0 (9.74) | | 0.227 | |
| **3** | 1.82 (3.34) | 2.78 (4.94) | 1.34 (2.28) | 3.37 (5.83) | 4.13 (6.95) | 4.92 (6.85) | 0.079 | 0.194 |
| **6** | 1.93 (3.90) | 2.19 (4.03) | 0.95 (1.59) | 1.28 (2.65) | 1.72 (2.10) | 3.74 (6.32) | 0.402 | **0.034** |
| **12** | 1.22 (2.84) | 1.93 (3.64) | 0.93 (1.80) | 1.03 (1.94) | 5.40 (6.79) | 3.93 (6.57) | 0.081 | **0.024** |

**Table S1: Treatment effectiveness**

Analysis of variances or Kruskal-Wallis test results revealed significant differences regarding treatment response in patients analysed per LOCF at 6 months and 12 months. Bonferroni corrected post hoc analysis revealed no significant differences after 6 months (guselkumab vs. risankizumab, *p* = 0.122; guselkumab vs. tildrakizumab, *p* = 0.667; risankizumab vs. tildrakizumab, *p* = 0.068), but there was a significant difference at 12 months for patients receiving risankizumab achieving a lower PASI compared to tildrakizumab (*p* = 0.041) (guselkumab vs. risankizumab, *p* = 0.126; guselkumab vs. tildrakizumab 0.46).

LOCF, last observation carried forward; PASI, Psoriasis Area and Severity Index; SD, standard deviation

|  |  |  |  | | |  |
| --- | --- | --- | --- | --- | --- | --- |
| **Treatment** | **Timepoint** | **Reduction category** | **Number (%) of patients** | | | ***p*-value** |
|  |  |  | **Naïve** | **Non-naïve** | **All** |  |
| **All treatments** | **3 months** | PASI 100 | 34 (44.2) | 17 (18.9) | 51 (30.5) | **0.0049** |
|  |  | >PASI 90 | 51 (66.2) | 28 (31.1) | 79 (47.3) |  |
|  |  | >PASI 75 | 60 (77.9) | 46 (51.1) | 106 (63.5) |  |
|  |  | >PASI 50 | 70 (90.9) | 69 (76.7) | 139 (83.2) |  |
|  |  | <PASI 50 | 7 (9.1) | 17 (18.9) | 24 (14.4) |  |
|  |  | increase | NA | 4 (4.4) | 4 (2.4) |  |
|  |  | All | 77 (100.0) | 90 (100.0) | 167 (100.0) |  |
|  | **6 months** | PASI 100 | 35 (60.3) | 16 (28.6) | 51 (44.7) | **0.0041** |
|  |  | >PASI 90 | 42 (72.4) | 22 (39.3) | 64 (56.1) |  |
|  |  | >PASI 75 | 50 (86.2) | 33 (58.9) | 83 (72.8) |  |
|  |  | >PASI 50 | 54 (93.1) | 40 (71.4) | 93 (81.6) |  |
|  |  | <PASI 50 | 4 (6.9) | 10 (17.9) | 14 (12.3) |  |
|  |  | increase | NA | 6 (10.7) | 6 (5.3) |  |
|  |  | All | 58 (100.0) | 56 (100.0) | 114 (100.0) |  |
|  | **12 months** | PASI 100 | 29 (65.9) | 14 (32.6) | 43 (49.4) | **0.0413** |
|  |  | >PASI 90 | 36 (81.8) | 19 (44.2) | 55 (63.2) |  |
|  |  | >PASI 75 | 39 (88.6) | 29 (67.4) | 68 (78.2) |  |
|  |  | >PASI 50 | 42 (95.5) | 33 (76.7) | 75 (86.2) |  |
|  |  | <PASI 50 | 1 (2.3) | 7 (16.3) | 8 (9.2) |  |
|  |  | increase | 1 (2.3) | 3 (7.0) | 4 (4.6) |  |
|  |  | All | 44 (100.0) | 43 (100.0) | 87 (100.0) |  |
| **Guselkumab** | **3 months** | PASI 100 | 19 (43.2) | 11 (17.2) | 30 (27.8) | 0.0187+ |
|  |  | >PASI 90 | 28 (63.6) | 15 (23.4) | 43 (39.8) |  |
|  |  | >PASI 75 | 33 (75.0) | 32 (50.0) | 65 (60.2) |  |
|  |  | >PASI 50 | 39 (88.6) | 48 (75.0) | 87 (80.6) |  |
|  |  | <PASI 50 | 5 (11.4) | 13 (20.3) | 18 (16.7) |  |
|  |  | increase | NA | 3 (4.7) | 3 (2.8) |  |
|  |  | All | 44 (100.0) | 64 (100.0) | 108 (100.0) |  |
|  | **6 months** | PASI 100 | 21 (56.8) | 13 (32.5) | 34 (44.2) | 0.0315+ |
|  |  | >PASI 90 | 25 (67.7) | 15 (37.5) | 40 (51.9) |  |
|  |  | >PASI 75 | 31 (83.8) | 21 (52.5) | 52 (67.5) |  |
|  |  | >PASI 50 | 34 (91.2) | 26 (65.0) | 60 (77.9) |  |
|  |  | <PASI 50 | 3 (8.1) | 10 (25.0) | 13 (16.9) |  |
|  |  | increase | NA | 4 (10.0) | 4 (5.2) |  |
|  |  | All | 37 (100.0) | 40 (100.0) | 77 (100.0) |  |
|  | **12 months** | PASI 100 | 17 (58.6) | 11 (35.5) | 28 (46.7) | 0.3094 |
|  |  | >PASI 90 | 22 (75.9) | 15 (48.4) | 37 (61.7) |  |
|  |  | >PASI 75 | 24 (82.8) | 22 (71.0) | 46 (76.7) |  |
|  |  | >PASI 50 | 27 (93.1) | 23 (74.2) | 50 (83.3) |  |
|  |  | <PASI 50 | 1 (3.4) | 6 (19.4) | 7 (11.7) |  |
|  |  | increase | 1 (3.4) | 2 (6.5) | 3 (5.0) |  |
|  |  | All | 29 (100.0) | 31 (100.0) | 60 (100.0) |  |
| **Risankizumab** | **3 months** | PASI 100 | 14 (58.3) | 6 (26.1) | 20 (42.6) | 0.6586 |
|  |  | >PASI 90 | 17 (70.8) | 13 (56.5) | 30 (63.8) |  |
|  |  | >PASI 75 | 21 (87.5) | 14 (60.9) | 35 (74.4) |  |
|  |  | >PASI 50 | 23 (95.8) | 21 (91.3) | 44 (93.6) |  |
|  |  | <PASI 50 | 1 (4.2) | 1 (4.3) | 2 (4.3) |  |
|  |  | increase | NA | 1 (4.3) | 1 (2.1) |  |
|  |  | All | 24 (100.0) | 23 (100.0) | 47 (100.0) |  |
|  | **6 months** | PASI 100 | 12 (75.0) | 3 (21.4) | 15 (50.0) | **< 0.0001** |
|  |  | >PASI 90 | 14 (87.5) | 7 (50.0) | 21 (70.0) |  |
|  |  | >PASI 75 | 16 (100.0) | 11 (78.6) | 27 (90.0) |  |
|  |  | >PASI 50 | 16 (100.0) | 13 (92.9) | 29 (96.7) |  |
|  |  | <PASI 50 | NA | NA | NA |  |
|  |  | increase | NA | 1 (7.1) | 1 (3.3) |  |
|  |  | All | 16 (100.0) | 14 (100.0) | 30 (100.0) |  |
|  | **12 months** | PASI 100 | 12 (85.7) | 3 (27.3) | 15 (60.0) | 0.2892 |
|  |  | >PASI 90 | 14 (100.0) | 4 (36.4) | 18 (72.0) |  |
|  |  | >PASI 75 | 14 (100.0) | 7 (63.6) | 21 (84.0) |  |
|  |  | >PASI 50 | 14 (100.0) | 10 (90.9) | 24 (96.0) |  |
|  |  | <PASI 50 | NA | 1 (9.1) | 1 (4.0) |  |
|  |  | increase | NA | NA | NA |  |
|  |  | All | 14 (100.0) | 11 (100.0) | 25 (100.0) |  |
| **Tildrakizumab** | **3 months** | PASI 100 | 1 (11.1) | NA | 1 (8.3) | **0.0013** |
|  |  | >PASI 90 | 6 (66.7) | NA | 6 (50.0) |  |
|  |  | >PASI 75 | 6 (66.7) | NA | 6 (50.0) |  |
|  |  | >PASI 50 | 8 (88.9) | NA | 8 (66.7) |  |
|  |  | <PASI 50 | 1 (11.1) | 3 (100.0) | 4 (33.3) |  |
|  |  | increase | NA | NA | NA |  |
|  |  | All | 9 (100.0) | 3 (100.0) | 12 (100.0) |  |
|  | **6 months** | PASI 100 | 2 (40.0) | NA | 2 (28.6) | 0.1303 |
|  |  | >PASI 90 | 3 (60.0) | NA | 3 (42.3) |  |
|  |  | >PASI 75 | 3 (60.0) | 1 (50.0) | 4 (57.1) |  |
|  |  | >PASI 50 | 4 (80.0) | 1 (50.0) | 5 (71.4) |  |
|  |  | <PASI 50 | 1 (20.0) | NA | 1 (14.3) |  |
|  |  | increase | NA | 1 (50.0) | 1 (14.3) |  |
|  |  | All | 5 (100.0) | 2 (100.0) | 7 (100.0) |  |
|  | **12 months** | PASI 100 | NA | NA | NA | 0.2231 |
|  |  | >PASI 90 | NA | NA | NA |  |
|  |  | >PASI 75 | 1 (100.0) | NA | 1 (100.0) |  |
|  |  | >PASI 50 | 1 (100.0) | NA | 1 (100.0) |  |
|  |  | <PASI 50 | NA | NA | NA |  |
|  |  | increase | NA | 1 (100.0) | 1 (100.0) |  |
|  |  | All | 1 (100.0) | 1 (100.0) | 2 (100.0) |  |

**Table S2: Achievement of treatment goals regarding previous biologic exposure**

Chi-square test results indicate statistically higher PASI 100, PASI > 90 and PASI > 75 responses in biologic-naïve patients.

Significant *p*-values are in bold. P-values <0.0167 were considered to be statistically significant due to Bonferroni correction.

NA not applicable (none); PASI, Psoriasis Area and Severity Index

| **Occurrence of adverse events** | **Number (%) of patients** | | |
| --- | --- | --- | --- |
|  | **All** | **Biologic-naive** | **Biologic-non-naïve** |
| **Yes** | 23 | 13 (56.52) | 10 (43.48) |
| **No** | 174 | 81 (46.56) | 93 (53.44) |

**Table S3: Occurrence of adverse events**

Numbers (percentages) of patients reporting an adverse event once regarding previous biologic exposure. A chi-square test result indicated no significant differences in occurrence of adverse events between biologic-naïve and -non-naïve patients (*p* = 0.368294).
